# Case Report: Schwann cell reprogramming and PDGF-driven nerve hypertrophy in an NF1 patient with CIDP-like autoimmunity

**DOI:** 10.3389/fimmu.2026.1862732

**Published:** 2026-07-17

**Authors:** Fei Wang, Wenqian Cao, Jiaye Lu, Run Huang, Yuhan Bai, Yining Zhang, Zilan Wang, Zhouqing Chen, Zhong Wang

**Affiliations:** 1 Department of Neurosurgery, The First Affiliated Hospital of Soochow University, Suzhou, Jiangsu, China; 2 Suzhou Medical College of Soochow University, Suzhou, Jiangsu, China

**Keywords:** chronic inflammatory demyelinating polyradiculoneuropathy (CIDP), hypertrophic neuropathy, neurofibromatosis type (NF1), PDGF signaling pathway, Schwann cell plasticity, single-cell transcriptomics

## Abstract

**Background and aims:**

Differentiating neoplastic proliferation from inflammatory fibrosis in peripheral nerve hypertrophy is critical. We report a patient with a neurofibromatosis type 1 (NF1) deletion exhibiting extreme diffuse nerve enlargement and chronic inflammatory demyelinating polyradiculoneuropathy (CIDP)-like autoimmunity. This study aims to elucidate the underlying endoneurial fibrotic mechanism, specifically focusing on the signaling networks between Schwann cells (SCs) and fibroblasts.

**Methods:**

Single-cell RNA sequencing was performed on a biopsied sural nerve to profile the cellular and transcriptomic landscape. Intercellular interactome and pseudotime trajectory analyses were utilized to map molecular evolution and signaling crosstalk.

**Results:**

Transcriptomic profiling revealed that SCs—which normally maintain myelin around axons and support peripheral nerve function—were pathologically entrapped in a dedifferentiated state. Serving as a genetic primer, the *NF1* deletion lowered the threshold for SC reprogramming, a vulnerability that was subsequently unleashed by a severe autoimmune infiltrate consisting of macrophages and T cells. These reprogrammed SCs abandoned myelin-maintaining genes, such as *MPZ*, to acquire a pro-fibrotic phenotype. Through a coordinated platelet-derived growth factor (PDGF) dual-axis network involving PDGFC-PDGFRA and PDGFD-PDGFRB, the entrapped SCs exclusively secreted PDGF ligands that potently activated endoneurial fibroblasts and vascular mural cells. This persistent paracrine signaling orchestrated excessive extracellular matrix deposition, driving a massive expansion of the endoneurial interstitium and the formation of classic “onion bulbs”.

**Interpretation:**

Our data suggest that the macroscopic hypertrophic changes observed in this specific clinical presentation may reflect an aberrant, immune-triggered fibrotic cascade—where autoimmune leukocyte infiltration continuously drives stromal overgrowth—complementing rather than entirely precluding the classical RAS/MAPK-driven neoplastic SC hyperproliferation. Furthermore, within the limitations of this pilot evaluation, characterizing this potential SC-fibroblast crosstalk indicates that the PDGF signaling pathway may warrant further investigation as a candidate translational therapeutic target for refractory hypertrophic neuropathies.

## Case description

A 51-year-old female presented with a progressive 3-year history of limb weakness, numbness, and severe muscle atrophy. In December 2022, following a respiratory infection, she developed widespread muscle fasciculations. Initial evaluation revealed generalized areflexia, “glove-and-stocking” sensory loss, and profound albuminocytologic dissociation, with cerebrospinal fluid (CSF) protein peaking at 3.44 g/L. Concurrently, whole-exome sequencing utilized on genomic DNA identified a pathogenic large heterozygous deletion at chromosome 17q11.2 involving exon 1 of the *NF1* gene. Bioinformatic analysis revealed a Variant Allele Frequency (VAF) of approximately 50% from peripheral blood, confirming a non-mosaic, constitutive germline mutation. Crucially, this large deletion was strictly confined to the *NF1* locus and did not encompass neighboring contiguous genes (such as *SUZ12* or *OMG*), indicating that the patient’s genotype does not reflect the classic, multi-gene 17q11.2 microdeletion syndrome. This genetic finding confirmed the diagnosis of Neurofibromatosis type 1 (NF1). Notably, classic NF1 cutaneous hallmarks, such as café-au-lait macules and iris Lisch nodules, were not identified by slit-lamp examination.

**Figure 1:**
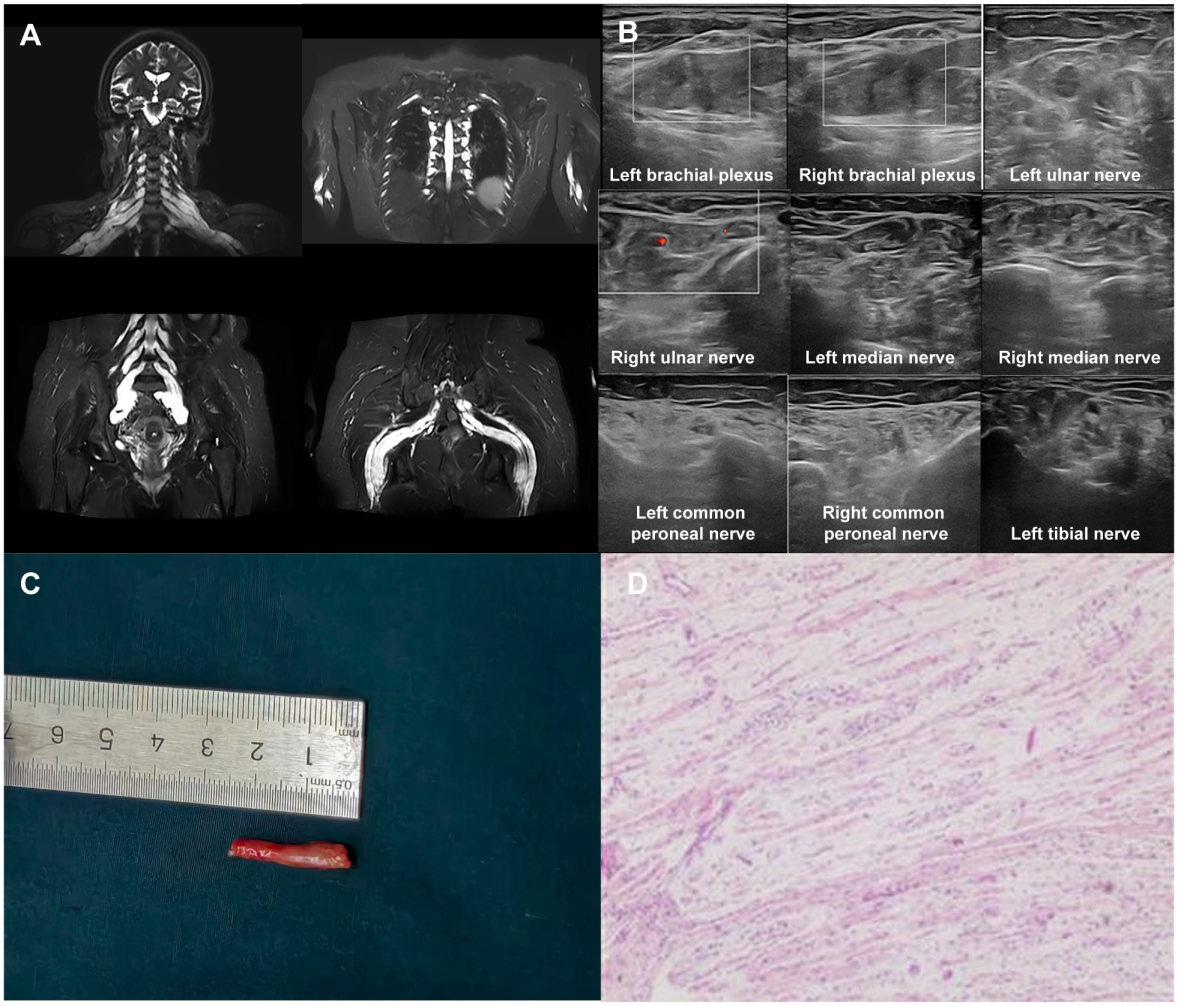
Clinical and pathological features of nerve hypertrophy. **(A)** Whole-body MRI (DWIBS) showing marked, symmetric hypertrophy and T2 hyperintensity of systemic nerve roots. **(B)** High-resolution ultrasound demonstrating massive enlargement of the ulnar, median, and brachial plexus nerves. **(C)** Macroscopic view of the resected left sural nerve during decompression surgery. **(D)** Histopathology of the sural nerve showing marked edema and myxoid degeneration, without neoplastic structures or amyloidosis.

By late 2023, electromyography confirmed a severe mixed demyelinating and axonal sensorimotor polyneuropathy. Advanced neuroimaging via whole-body diffusion-weighted imaging with background body signal suppression (DWIBS) and 3D reconstruction demonstrated marked, symmetric hypertrophy of systemic nerve roots exhibiting prominent T2 hyperintensity (**Figure 1A**). Consistent with these radiologic findings, high-resolution ultrasound further quantified the massive, diffuse enlargement of peripheral nerves, including the ulnar (maximum diameter: 14.4 mm), median (13.3 mm), and brachial plexus trunks (11.6 mm, **Figure 1B**). When benchmarked against established normative values from healthy controls—where typical adult peripheral nerve diameters consistently range between 2.0 mm and 3.5 mm across these specific segments—the patient’s nerve diameters reflect an extraordinary 4-to-5-fold increase over the upper limits of normal, quantitatively establishing the unprecedented scale of this systemic hypertrophic neuropathy. Regarding the ^18^F-FDG PET-CT findings, the quantitative analysis demonstrated only mild glucose tracer uptake within the thickened bilateral lumbar, sacral, and pelvic wall nerves, with a maximum standardized uptake value (SUVmax) of 2.96. Given that malignant peripheral nerve sheath tumors (MPNSTs) characteristically exhibit intense glucose hypermetabolism with SUVmax values typically well exceeding 5.0–10.0, this low quantitative metric strongly supports that the diagnosis of MPNST is highly unlikely.

A therapeutic trial with selumetinib—an allosteric MEK1/2 inhibitor designed to suppress the downstream RAS/RAF/MEK/ERK cascade and arrest NF1-driven neoplastic proliferation (1, 2)—proved completely ineffective. Conversely, subsequent cranial nerve involvement responded temporarily to high-dose corticosteroids and intravenous immunoglobulin (IVIG), highlighting a distinct “therapeutic dissociation.” This pattern strongly suggested the extreme neural hypertrophy was driven by an autoimmune process akin to chronic inflammatory demyelinating polyradiculoneuropathy (CIDP) with secondary inflammatory edema, rather than classical RAS/MAPK-driven neoplastic SC hyperproliferation.

In August 2025, severe secondary multi-site nerve entrapment necessitated extensive peripheral nerve decompression alongside a left sural nerve biopsy (**Figure 1C**). Histopathological analysis revealed marked nerve fiber edema and myxoid degeneration without neurofibromatous neoplastic structures. Special stains for amyloidosis (Congo red staining) and acid-fast bacilli were strictly negative (**Figure 1D**). Postoperatively, the patient was discharged on a maintenance immunomodulatory regimen of mycophenolate mofetil (250 mg BID), yielding significant attenuation of cervicobrachial pain and improved limb mobility. From a rigorous diagnostic standpoint, the patient fully satisfied the formal European Academy of Neurology/Peripheral Nerve Society (EAN/PNS) 2021 diagnostic criteria for CIDP, established by the progressive sensorimotor deficits lasting over two months, demyelinating electrodiagnostic features, and profound CSF albuminocytologic dissociation. To rigorously differentiate this from an overlapping inherited neuropathy, a comprehensive re-evaluation of the whole-exome sequencing data was performed, which confirmed the absolute absence of pathogenic variants in canonical Charcot-Marie-Tooth disease-related genes (e.g., *PMP22*, *MPZ*, *GJB1*, *MFN2*). Furthermore, commercial screening for canonical seropositive nodal/paranodal autoantibodies (including anti-NF155, anti-CNTN1, and anti-CASPR1) returned negative or inconclusive results. This specific diagnostic profile—fully meeting inflammatory demyelinating criteria but lacking classical autoantibodies—led us to define this presentation precisely as a “CIDP-like” autoimmune manifestation rather than an atypical variant of pure NF1-associated hypertrophy.

### Sural nerve scRNA-seq profiling

To decipher the molecular landscape driving this massive neural hypertrophy, the biopsied fresh sural nerve tissue was subjected to single-cell RNA sequencing (scRNA-seq) utilizing the 10x Genomics platform. After stringent quality control, a total of 20,682 high-quality cells were retained for downstream analysis. Unsupervised clustering initially yielded 16 distinct cell clusters, which were subsequently annotated into seven primary cell lineages: Fibroblasts,SCs, Macrophages, Endothelial cells, Mural cells, T cells, and Mast cells. Notably, a pronounced stromal expansion was quantitatively reflected by the overwhelming dominance of fibroblast-like cells (n=10,023) over the SC population (n=4,572).

**Figure 2:**
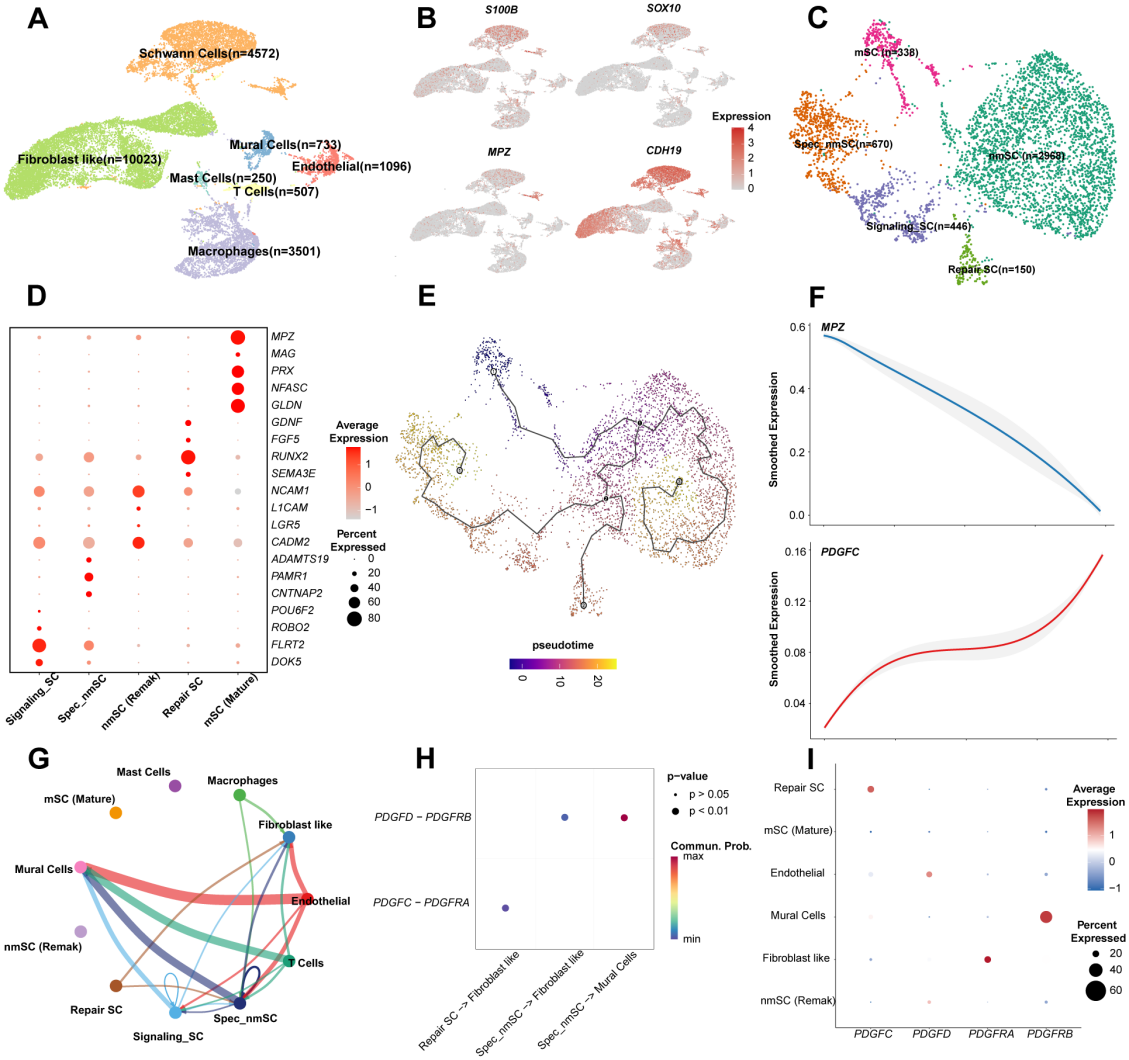
Single-cell atlas and PDGF signaling in hypertrophic nerves. **(A)** UMAP plot of 20,682 cells revealing seven major cell lineages. **(B)** Expression distribution of key Schwann cell (SC) markers (*SOX10*, *MPZ*, *S100B*, *CDH19*) across clusters. **(C)** Sub-clustering of the SC lineage into five distinct functional states. **(D)** Expression profile of specific marker genes identifying the SC subpopulations. **(E)** Pseudotime trajectory showing SC evolution from a mature state (mSCs) to a dedifferentiated Repair SC state. **(F)** Gene expression dynamics in pseudotime, highlighting the decline of *MPZ* and terminal upregulation of *PDGFC*. **(G)** Cell-cell communication network highlighting the dual-axis PDGF signaling. **(H)** Bubble plot of significant ligand-receptor pairs, specifically the *PDGFC-PDGFRA* and *PDGFD-PDGFRB* axes mediating SC-stromal crosstalk. **(I)** Expression mapping of key communication factors driving the fibrotic cascade.

To further untangle the nerve-specific alterations, sub-clustering of the SC lineage was performed, delineating five distinct cellular states: Remak SCs (non-myelinating SCs [nmSCs] that surround and support multiple small peripheral axons), specialized nmSCs, signaling SCs, mSCs, and Repair SCs (**Figures 2A–D**). Pseudotime trajectory analysis of the scRNA-seq data delineated a continuous, directional evolutionary trajectory originating from mSCs and terminating at a distinct subset of Repair SCs (**Figure 2E**). At the transcriptomic level, this switch was characterized by a precipitous decline in the expression of the myelin-maintaining gene *MPZ*, which precisely mirrored the eruptive, terminal expression of the pro-fibrotic ligand *PDGFC* (**Figure 2F**). Furthermore, intercellular interactome analysis mapped a robust, coordinated dual-axis PDGF signaling network within the microenvironment: the Repair SC subset exclusively secreted PDGFC to target PDGFRA on endoneurial fibroblasts, while specialized nmSCs utilized the PDGFD-PDGFRB axis to engage vascular mural cells (**Figures 2G–I**).

## Discussion

Diffuse peripheral nerve enlargement in an *NF1* patient typically triggers an immediate clinical assumption of plexiform neurofibromas, often prompting oncological interventions (3, 4). However, this case illustrates a critical diagnostic pitfall: a superimposed autoimmune insult can hijack a pre-existing genetic susceptibility to drive anomalous, non-neoplastic tissue remodeling. By applying single-cell transcriptomic resolution, we redefine these macroscopic hypertrophic changes not as RAS-driven tumor proliferation, but as an aberrant, immune-triggered fibrotic cascade operating under an *NF1*-deficient background.

### Schwann cell plasticity and pathological entrapment

Following inflammatory demyelination, mature SCs (mSCs) possess a remarkable physiological plasticity (5, 6), typically dedifferentiating into a transient “Repair SC” state to clear myelin debris, secrete neurotrophic factors, and guide axonal regeneration (7, 8). Pseudotime trajectory analysis of our scRNA-seq data delineated a continuous, directional evolution from mSCs to a distinct subset of Repair SCs (**Figure 2E**). However, the SCs in this patient appeared pathologically “entrapped” in this dedifferentiated state, failing to transition back into a myelinating phenotype. This chronic transcriptional arrest is robustly supported by the striking and absolute divergence within our dataset: a precipitous decline in the myelin-maintaining gene *MPZ* precisely mirrored the eruptive, terminal expression of the pro-fibrotic ligand *PDGFC* (**Figure 2F**). This persistent, locked phenotype draws strong precedence from established clinical paradigms of chronic autoimmune and demyelinating neuropathies. Specifically, it has been demonstrated that autoantibody-mediated insults can directly augment SC mitotic activity while suppressing the master myelination regulator *Krox20*, effectively freezing the cells in a non-myelinating, proliferative state (9). Furthermore, classical histopathological evidence confirms that such persistent demyelinating stress continuously drives SC hyperproliferation, which is invariably coupled with a massive expansion of endoneurial and perineurial collagen (10). Notably, even mild demyelinating pathophysiology is capable of chronically engaging SCs to orchestrate onion-bulb remodeling (11).

We postulate a “two-hit” paradigm for this phenotypic entrapment, wherein an intrinsic genetic vulnerability cooperates with an immunologically driven injury microenvironment. Specifically, *NF1* haploinsufficiency acts as the primary genetic primer (Hit 1), which dysregulates intracellular signaling and lowers the threshold for SC reprogramming without autonomously triggering hypertrophic overgrowth (12). This latent vulnerability is actively unleashed by a secondary autoimmune insult (Hit 2)—the CIDP-like process—which behaves as a chronic “nodo-paranodopathy” that directly disrupts axo-glial and SC-axonal interactions at the nodal and paranodal regions (13). While such axonal-glial dissociation normally triggers a transient, self-limiting repair state, in this *NF1*-deficient genetic background it establishes a persistent “injury microenvironment” that completely blocks the feedback loops required for SC re-differentiation (12). Deprived of stabilizing axonal constraints, these primed SCs “escape” the normal regenerative pathway and become pathologically locked in a toxic, dedifferentiated “Repair SC” phenotype, chronically driving the downstream *PDGFC*-mediated stromal expansion and macroscopic, rope-like neural hypertrophy.

Mechanistically, the molecular identity of this upstream autoimmune trigger is corroborated by both our transcriptomic data and the patient’s clinical course. Our scRNA-seq profiling directly identifies a conspicuous inflammatory infiltrate consisting of endoneurial Macrophages, T cells, and Mast cells within the hypertrophic microenvironment. In the context of antibody-mediated chronic nodo-paranodopathies, the persistent disruption of the axo-glial architecture acts as a potent chemoattractant, driving the chronic recruitment and activation of macrophages and T lymphocytes (14, 15). These infiltrating leukocytes release a localized cascade of pro-inflammatory cytokines and fibrotic rheostats—including TNF-*α*, IL-1 *β*, and macroenvironment-derived TGF-*β*—which have been shown to experimentally cooperate with *NF1* deficiency to accelerate cellular proliferation and prevent myelin gene re-expression (12). This chronic neuro-inflammatory axis effectively creates the sustained “injury microenvironment” that locks Repair SCs into their anomalous, pro-fibrotic secretory state. Clinically, the validity of this upstream immune drive is strongly supported by the patient’s favorable therapeutic response to targeted immunomodulation via corticosteroids and mycophenolate mofetil, which arrested the demyelinating cascade by directly suppressing this leukocyte-driven inflammatory circuit.

### The PDGFC/D axis and extracellular matrix expansion

The histological hallmark of chronic hypertrophic neuropathies is the “onion bulb”—concentric (16), multilayered whorls of supernumerary SCs and fibroblasts tightly encircling hypomyelinated axons. Our intercellular interactome analysis maps the precise molecular blueprint of this pathognomonic phenomenon. By isolating the PDGF pathway within the global cell-cell communication network (**Figure 2G**), we uncovered a putative, spatially coordinated dual-axis signaling architecture that is bioinformatically inferred to underlie this structural remodeling. Specifically, the entrapped Repair SCs preferentially utilize the PDGFC–PDGFRA axis to directly engage endoneurial fibroblasts, while specialized nmSCs simultaneously leverage the PDGFD–PDGFRB cascade to target vascular mural cells (**Figures 2H, I**). This relentless, bidirectional paracrine signaling orchestrates a massive expansion of the endoneurial interstitium through excessive collagen and extracellular matrix deposition, conceptually aligning with the profound numerical dominance of fibroblasts over the SC lineage captured in our dataset. Consequently, we propose a model wherein these reprogrammed SCs act as permanent “pathogenic anchors”. Under this framework, the continuous recruitment and activation of periaxonal fibroblasts by these altered glia may serve as the primary driver behind the macroscopic, rope-like nerve hypertrophy identified on clinical neuroimaging. We explicitly note that these intercellular networks represent computational predictions generated from transcriptional ligand-receptor transcripts, and further prospective *in vitro* or *in vivo* experimental validations are required to definitively establish these as functional signaling events.

### Translational implications for targeted therapy

These molecular insights support the hypothesis of a potential mechanistic link underlying the patient’s unique clinical course and therapeutic refractoriness. While a baseline contribution of NF1-associated RAS/MAPK signaling cannot be excluded—and likely served as the foundational genetic primer—the profound endoneurial stromal expansion and extracellular matrix deposition observed in our dataset suggest that the macroscopic neural hypertrophy may be predominantly driven by an advanced, downstream immune-associated fibrotic cascade rather than classical pure neoplastic hyperproliferation alone. Consequently, the lack of clinical response to the MEK inhibitor selumetinib may not imply the total absence of RAS/MAPK involvement; rather, it could reflect that the established fibrotic tissue remodeling is non-reversible through pure MEK blockade, or alternatively, it may represent intrinsic treatment resistance emerged from the complex, inflammatory microenvironment. Instead, mitigating this parallel upstream autoimmune trigger required targeted immunomodulation (e.g., mycophenolate mofetil and corticosteroids) to suppress the ongoing demyelinating insults.

Furthermore, identifying the *PDGFC/D* signaling hub uncovers a highly promising therapeutic vulnerability. For hypertrophic neuropathies refractory to standard immunotherapies, repurposing specific PDGF receptor tyrosine kinase inhibitors (such as imatinib, which has demonstrated potent anti-fibrotic efficacy in hepatic and renal models) could effectively interrupt the pathological SC-fibroblast crosstalk (17)—specifically, the aberrant signaling between Repair SCs and endoneurial fibroblasts, as well as between specialized nmSCs and vascular mural cells. This targeted approach offers a preliminary, hypothesis-generating framework for these tyrosine kinase inhibitors, representing a candidate pharmacological strategy that warrants future prospective validation in specialized preclinical models prior to any translational consideration.

Nevertheless, we acknowledge that establishing a definitive, unidirectional causal chain from a single-case observation has inherent limitations. Future studies—including mechanistic functional assays, larger clinical cohort validations, or prospective experimental models—are required to further elucidate the precise sequential relationships and molecular cross-talk between this CIDP-like autoimmune process, SC reprogramming, localized tissue fibrosis, and the profound neural hypertrophy observed in this patient. Additionally, while these single-cell transcriptomic findings provide valuable insights based on canonical marker definitions, the lack of a direct healthy human sural nerve control dataset—owing to the severe ethical and clinical constraints of obtaining normal nerve biopsies—remains an inherent limitation of this single-case report. Consequently, subsequent validation across expanded clinical repositories or specialized animal models remains essential to fully delineate the precise molecular kinetics of this process.

## Conclusion

In conclusion, our findings suggest that macroscopic peripheral nerve hypertrophy in the context of *NF1* deficiency and a superimposed CIDP-like autoimmunity may be driven by an aberrant, immune-triggered fibrotic cascade, complementing rather than entirely precluding classical neoplastic proliferation. Our single-cell transcriptomic data support the hypothesis that reprogrammed SCs potentially act as pathogenic anchors, contributing to endoneurial stromal expansion via a putative dual-axis PDGF signaling network. Clinically, identifying this potential pathomechanism may help prevent inappropriate oncological interventions; furthermore, our bioinformatic findings suggest that PDGF signaling may represent a potential therapeutic target warranting further experimental investigation. Nevertheless, within the limitations of a single-case observation lacking a healthy control nerve dataset and direct functional assays, these computational predictions remain preliminary and warrant future experimental validation in larger clinical cohorts and targeted animal models.

## Data Availability

The raw data supporting the conclusions of this article will be made available by the authors, without undue reservation.
